# Establishing and evaluating the gradient of item naming difficulty in post-stroke aphasia and semantic dementia

**DOI:** 10.1016/j.cortex.2024.07.007

**Published:** 2024-10

**Authors:** Erling Nørkær, Ajay D. Halai, Anna Woollams, Matthew A. Lambon Ralph, Rahel Schumacher

**Affiliations:** a Department of Psychology, University of Copenhagen, Copenhagen, Denmark; b MRC Cognition and Brain Sciences Unit, University of Cambridge, Cambridge, United Kingdom; c Division of Neuroscience and Experimental Psychology, University of Manchester, Manchester, United Kingdom; d Clinic for Neurology and Neurorehabilitation, Luzerner Kantonsspital, University Teaching and Research Hospital, and University of Lucerne, Lucerne, Switzerland

**Keywords:** Naming, Item response theory, Post-stroke aphasia, Semantic dementia

## Abstract

Anomia is a common consequence following brain damage and a central symptom in semantic dementia (SD) and post-stroke aphasia (PSA), for instance. Picture naming tests are often used in clinical assessments and experience suggests that items vary systematically in their difficulty. Despite clinical intuitions and theoretical accounts, however, the existence and determinants of such a naming difficulty gradient remain to be empirically established and evaluated. Seizing the unique opportunity of two large-scale datasets of semantic dementia and post-stroke aphasia patients assessed with the same picture naming test, we applied an Item Response Theory (IRT) approach and we (a) established that an item naming difficulty gradient exists, which (b) partly differs between patient groups, and is (c) related in part to a limited number of psycholinguistic properties - frequency and familiarity for SD, frequency and word length for PSA. Our findings offer exciting future avenues for new, adaptive, time-efficient, and patient-tailored approaches to naming assessment and therapy.

## 1 Introduction

Anomia – problems in naming objects and concepts – is a common consequence following brain damage of numerous aetiologies. It is one of the central symptoms in semantic dementia and post-stroke aphasia, for instance (Hodges & Patterson, 2007; Kohn & Goodglass, 1985; Lambon Ralph et al., 2001; Woollams et al., 2008). Hence, naming tests are often part of a clinical evaluation in various patient groups. Clinical experience strongly suggests that there are systematic variations across items, making certain items harder to name than others. These systematic differences in item naming difficulty are also implied in classical models of naming, e.g., reflected as different activation thresholds in Morton's (1969) logogen model or in the model of lexical access in picture naming by Dell et al. (1997). Despite strong clinical intuitions and theoretical accounts, however, the existence and determinants of such a naming difficulty gradient remain to be empirically established and evaluated. Seizing the unique opportunity of two large-scale datasets of semantic dementia and post-stroke aphasia patients assessed with the same picture naming test, we applied an Item Response Theory (IRT) approach to explore: (a) if there are systematic differences in item naming difficulty, (b) if the naming difficulty gradient is different across aetiologies, and (c) how much these item difficulty gradients relate to psycholinguistic word properties. Establishing the presence and nature of a systematic gradient of item naming difficulty would allow for new approaches to systematic test construction and adaptive, time-efficient assessments tailored as needed for each patient group, as well as unlock new approaches to naming therapy that utilize the difficulty gradients to create “zone of proximal re-development” rehabilitation programmes (Conroy et al., 2012; Vygotsky, 1978).

### 1.1 Establishing a systematic gradient of item naming difficulty

Many different naming tests exist and some were developed to contain items of varying difficulty. Among the most widely used examples are the Graded Naming Test (GNT; Mckenna & Warrington, 1983) and the Boston Naming Test (BNT; Goodglass et al., 1983) in English, and the naming test of the Aachener Aphasie Test (AAT; Huber et al., 1983) in German. However, little research has evaluated whether the variation of item difficulty aligns with patients’ naming success and if this is the same across groups.

Given a sufficiently large and appropriate dataset, one formal approach to establishing the presence and nature of a difficulty gradient in patients' performance is IRT. IRT provides a powerful statistical framework for deriving psychometric properties of individual items while at the same time taking into account an individual's severity/ability (Thomas, 2019). The core principle in IRT is to derive a mathematical model that estimates one or more parameters pertaining to each item using individual item response results (Rasch, 1960). By adopting an IRT approach, we can ask whether items vary in naming difficulty, and whether the items discriminate well between individuals across different levels of anomia severity.

One of the few previous studies employed IRT to investigate the item parameters of the BNT in a sample of 300 (non-aphasic) patients (Pedraza et al., 2011). Similarly, in a study of 69 patients with mild Alzheimer's disease, Graves et al. (2004) adopted an IRT approach to derive item parameters for the BNT with the aim of comparing different short versions of the test. While these studies do address the question of whether an item difficulty gradient exists, they were performed with participants who did not (at least not necessarily) present with naming problems and therefore cover only a limited range of possible anomia severity.

### 1.2 The influence of aetiology on the item naming difficulty gradient

If item naming difficulty gradients do exist, it is relevant to evaluate if they are the same across patient groups. The importance of the question is twofold. First, it is important to determine whether it is reasonable to model item parameters so that they are freely estimated across patients with different aetiologies. This is only warranted if it can safely be assumed that items are equally difficult for these different patient groups. Second, *if* some items are more difficult for one patient group than another regardless of the level of anomia severity it may suggest the need for tailored assessment tools for different patient cohorts. One way to investigate whether item difficulty differs between aetiologies is by means of analysis of differential item functioning (DIF), an IRT based method that assesses whether the difficulty (and/or discrimination) gradient varies between groups (Chalmers et al., 2016; Teresi et al., 2021).

### 1.3 Relating the difficulty gradient to item properties

If a systematic gradient of item naming difficulty can be established, it is of interest to explore how much of this item difficulty gradient is related to different factors (e.g., psycholinguistic properties). Furthermore, if the gradient appears to be different between aetiologies, this might also be reflected in a different pattern of relevant factors. Past work has tackled elements of this two-part question. An older literature looked directly at the relationship of item properties to naming success in post-stroke aphasia (Ellis et al., 1996; Nickels & Howard, 1995) and semantic dementia (Lambon Ralph et al., 1998) but did not establish the gradient of item naming difficulty itself or compare these variables directly across the groups. In more recent research, Fergadiotis et al. (2015, 2019) used IRT to establish a naming gradient in PSA on the Philadelphia Naming Test and related this to psycholinguistic item properties, but did not explore how this ‘psycholinguistic makeup’ differed between aetiologies.

Given that the underpinning cause of naming difficulties in these two patient groups is different, we might expect deviations not only in their item difficulty gradients but also in any relationship with psycholinguistic properties. Specifically, the anomia in SD appears to result from the gradual dissolution of the underlying conceptual-semantic representations (Lambon Ralph et al., 2001; Woollams et al., 2008), and item properties relating to semantics, such as familiarity, frequency and age of acquisition have been found to influence naming success (Lambon Ralph et al., 1998). Anomia in PSA, on the other hand, most commonly reflects a primary phonological impairment plus variable levels of semantic control weakness (Lambon Ralph et al., 2002; Schwartz et al., 2006). Therefore, not only frequency and age of acquisition but also word length has been documented to influence naming success (Ellis et al., 1996; Fergadiotis et al., 2015; Nickels & Howard, 1995, 2004).

To our knowledge, the full two-part comparative exploration (establishing the relationship between a gradient of item naming difficulty and (psycholinguistic) item properties, and then exploring how it differs between aetiologies) remains to be achieved. The answer is important not only for advancing the understanding of the bases of naming impairments, but also because it potentially unlocks new approaches to naming assessment and therapy based on effective and efficient sampling of the relevant item properties.

## 2 Methods

### 2.1 Participants

This study analysed data from two large samples of patients with two different aetiologies: a sample of 80 patients with chronic post-stroke aphasia (PSA), reported for instance in Halai et al. (2020) plus some new cases recruited with the same inclusion criteria (first-ever, left-sided stroke, at least twelve months prior to inclusion; right-handed native English speakers; any aphasia type or severity), and a sample of 67 patients diagnosed with semantic dementia (SD) (Woollams et al., 2008) who were assessed longitudinally (yielding a total of 160 observations). Given the significant decline between testing sessions, the longitudinal SD data were treated as independent observations in the analyses, in line with previous publications (Woollams et al., 2008). Table 1 contains characteristics of each sample. Informed consent was obtained from all participants prior to participation, in line with the Declaration of Helsinki and as approved by the local NHS ethics committee.

**Table 1:** Characteristics of the two samples.

			CSB Naming Performance			
Patient group	N	Mean age	Mean	SD	Min	Max
Semantic Dementia	160	62.8	25.44	19.91	0	64
Post-Stroke Aphasia	80	65.2	32.17	21.25	0	61

CSB: Cambridge Semantic Memory Test Battery, N: Number of patients/observations in that sample.

### 2.2 Measures

All patients were administered the naming test of the Cambridge Semantic Memory Test Battery (CSB), a set of tests used to assess semantic knowledge across different modalities in a clinical setting (Adlam et al., 2010; Bozeat et al., 2000). The naming test contains 64 black and white line drawings of common objects selected to cover 8 different semantic categories with 8 items each: domestic and foreign animals, birds, fruits, small and large household items, vehicles and tools. The object drawings were taken from the Snodgrass and Vanderwart (1980) 260 standardized picture set. The performance of both patient groups spanned not only the full range of possible scores (as shown in Table 1), but was also evenly distributed within both groups which makes the dataset optimally suited for an IRT approach: as explained below, IRT simultaneously models item difficulty and participant performance, and thus the IRT estimates of these two parameters is best when the sample (for both groups) covers the full range of scores.

### 2.3 Statistical analyses

#### 2.3.1 Item analyses

**Fig. 1:**
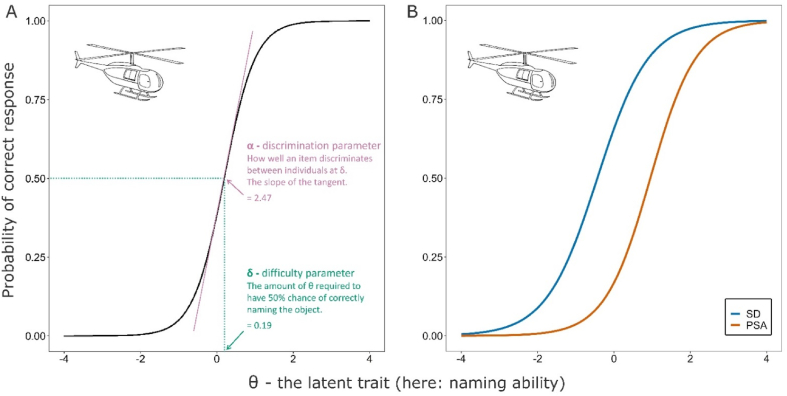
*Item characteristic curves (ICC) to illustrate the basics of IRT (A) and an example of DIF (B).* A: ICC of the item *helicopter*. The parameters visualized here are derived from the Constrained Model, in which item parameters are constrained to be equal across all patients. B: ICC of the item *helicopter* to illustrate DIF. The parameters visualized here are derived from the Final Model. For PSA patients, the drawing of a helicopter requires more of the underlying trait to accurately name (*δ* = .95) than for SD patients (*δ* = −.44).

IRT is a powerful tool in estimating psychometric properties of clinical assessments in that it models patient severity alongside item difficulty (Thomas, 2019). To assess the item parameters of the CSB naming test, a set of unidimensional two parameter logistic models were fitted to the dichotomous response data (naming success yes/no) of the two patient samples. All IRT modelling was conducted using the mirt R package (Chalmers, 2012). The modelling resulted in the derivation of two distinct parameters for each item. Fig. 1A illustrates the item parameters of *helicopter*. The item *difficulty parameter* indicates the amount of the underlying trait, theta, required for a 50 % probability of correctly naming that object. The item *discrimination parameter* is the slope of the tangent at the point of inflection of the item characteristic curve and indicates how well an item discriminates between individuals at that level of theta. Individual person parameters (theta values for each patient) can be easily derived from the item parameters and individual patient test performance. As the IRT model is essentially a unidimensional confirmatory factor analysis, the theta values are simply individual factor scores on the extracted factor.

Initially, a Constrained Model was fitted with item parameters restricted to be equal across the two patient samples. If one expected the item parameters to be the same regardless of aetiology, this initial model could be sufficient. However, as one goal of this study was to investigate how items differed in difficulty between patient populations, the Constrained Model was used as a baseline for further analyses. From the Constrained Model, a set of anchor items for the subsequent DIF analyses was determined based on a procedure outlined by Meade and Wright (2012). Potential anchors are identified by assessing each item for potential DIF by allowing the target item's parameters to vary freely while keeping all other items constrained as anchor items (cf. Kopf et al. (2015) for different strategies in anchor item selection). From the resulting set of identified non-DIF items, the five items with the highest discrimination parameter values were chosen as anchor items for fitting an Anchored Model. The five items were *pineapple, saw, scissors, swan* and *candle.* In the Anchored Model, these five items were constrained to have equal parameters across samples, and DIF for the remaining 59 items was investigated. Lastly, a Final Model was fitted using all non-DIF items from the preceding DIF analysis as anchor items. The rationale for this Final Model was that if items did not show substantial DIF, their parameters should not be allowed to vary freely between patient groups. Therefore, item parameters for all non-DIF items were constrained to possess identical item parameter values for each patient group.

All IRT models were fitted using an expectation-maximization algorithm with fixed quadrature (Chalmers, 2012). The Constrained Model and the Anchored Model were fitted using a Gaussian prior distribution of the latent trait, whereas the Final Model was fitted using the empirical histogram method for specifying the prior distribution of the latent trait as described by Bock and Aitkin (1981). To assess the assumption of unidimensionality, the following model fit indices were calculated and evaluated: Tucker–Lewis Index (TLI), Comparative Fit Index (CFI) and Root Mean Square Error of Approximation (RMSEA). The fit indices were evaluated against the cut-off values recommended by Hu and Bentler (1999).

#### 2.3.2 Regression analyses

In order to investigate which aspects of an item influence its difficulty and discrimination, a set of linear multiple regressions was computed for the two patient samples separately. The aim of these analyses was to determine which psycholinguistic and other variables were significantly related to the item parameters, and to what extent. Item difficulty or discrimination, respectively, were the dependent variables and the following independent variables were included: 1) word length, 2) frequency (HAL study, Lund and Burgess (1996)), 3) age of acquisition (Kuperman et al., 2012), 4) semantic diversity, a computed measure of how much a word's meaning varies across contexts (Hoffman et al., 2013), 5) familiarity, a rated measure of how often each concept is encountered (Rossion & Pourtois, 2004), and 6) naming reaction time (RT) in healthy controls (Torrance et al., 2018). Given the core research questions in this study, we selected, a priori*,* the variables that have previously been shown to be most important in at least one of the two patient groups (see Introduction). Healthy participant naming time was also added because it has been shown to be a partial predictor of item naming gradients in one previous study (Fergadiotis et al., 2019). Variables 1–3 were obtained from the English Lexicon Project Web Site (Balota et al., 2007), semantic diversity from the study by Hoffman et al. (2013), familiarity from the normative data collection study by Rossion and Pourtois (2004), and naming RT in healthy controls from the Multilanguage Written Picture Naming Dataset (MWPND; Torrance et al., 2018). Since some items, e.g., *lorry*, have several competing correct answers (*lorry*, *truck*), the respective values were extracted from the word that was most commonly given as an answer according to the MWPND (Torrance et al., 2018). One item consists of two words (*watering can*) and was not part of the English Lexicon Project, therefore the regressions are based on data for 63 items only.

Additionally, a follow-up regression analysis with Group (SD vs. PSA) and all Group x Item property interaction terms was conducted. The rationale for this analysis was that if an item property appeared to have substantially different importance for an item parameter between the two patient groups, then the difference needed to be formally assessed via the Group x Item property interaction. In the end, this follow-up analysis was only carried out for item difficulty as the regressions with item discrimination as dependent variable performed too poorly (see Results section for more details).

### 2.4 Availability of data and analysis code

The conditions of our ethics approval do not permit public archiving of anonymised study data. Readers seeking access to the PSA data should contact Prof. Lambon Ralph. Access will be granted to named individuals in accordance with ethical procedures governing the reuse of sensitive data and after completion of a formal data sharing agreement. The data included in the regression analyses as well as the code for the IRT and multiple regression analyses can be found here: https://osf.io/t732n. No part of the study procedures or analyses was pre-registered prior to the research being conducted.

## 3 Results

### 3.1 Item analyses

Our first two aims were to investigate if a gradient of item naming difficulty could be established and whether this gradient varied between different aetiologies (PSA vs. SD). To this end, a series of IRT models was fitted to the individual response data of the two patient groups, resulting in a Final Model with two sets of item parameters (difficulty and discrimination) per group. The fit indices (RMSEA = .03, CFI = .99 and TLI = .99) of the Final Model indicated an excellent fit to a unidimensional structure when comparing these values to the recommended cut-offs by Hu & Bentler (1999). This result suggests that the CSB naming test measures one underlying trait and licenses the further analysis of individual item parameters.

**Fig. 2:**
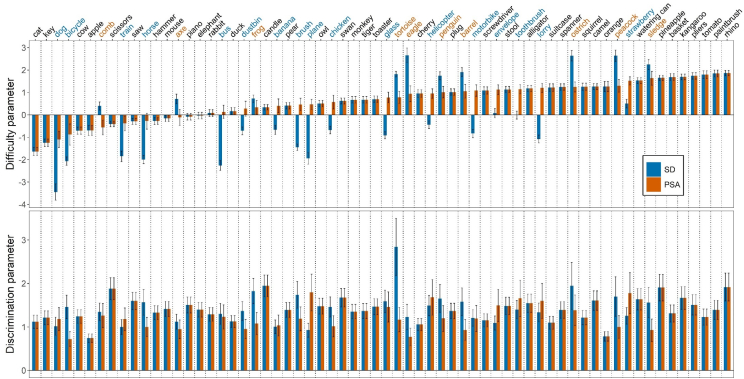
*Difficulty and discrimination parameters of each item for the Final IRT models.* Items are sorted by increasing difficulty based on the PSA item parameters. Error bars indicate Standard Error of the estimated item parameter. Coloured words indicate that the DIF was significant. Blue-coloured words indicate that the item is systematically easier for SD than for PSA patients, orange-coloured words vice versa.

Even though the items were widely distributed in terms of item difficulty for both patient group models, the gradient differed between the two clinical populations. These differences can be inspected in the upper panel of Fig. 2 – *helicopter*, for instance, contains a higher difficulty parameter in the PSA (*δ* = .95) than in the SD (*δ* = −.44) sample. Similarly, items discriminated well across different levels of the underlying trait, but between-group differences in discrimination power were also present, as shown in the lower panel of Fig. 2 (discrimination parameter, *α*, for *helicopter* was 1.68 and 1.49 for the PSA and SD sample, respectively).

The between-group disparities in item parameters can be formally investigated by looking at which items show DIF. Fig. 1B shows the ICC curves for our example item (*helicopter*) which is one of the items that shows DIF. Thus, given the same level of naming impairment, *helicopter* is systematically easier to name for an individual with SD *vs* an individual with PSA. In total, DIF was significant in 27 items (10 in favour of PSA, 17 in favour of SD patients). This suggests that approximately half of the items’ parameters differ across the two patient groups. More details on the individual item parameters can be found in the Supplementary Table S1.

### 3.2 Regression analyses

**Fig. 3:**
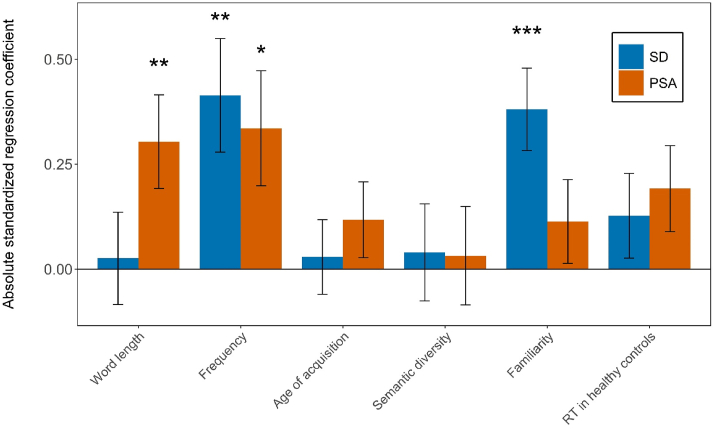
*Absolute standardized regression coefficients of the regression models on item difficulty for both patient groups.* Coefficients are absolute values. Asterisks (∗, ∗∗, ∗∗∗) indicate significant difference from zero at α = .05, .01 and .001 levels, respectively. Error bars indicate Standard Error of the Estimate.

Next, we characterized the extent to which psycholinguistic and other variables contribute to an item's naming difficulty and discrimination as well as to elucidate possible group differences in these patterns of relevant factors. Linear multiple regressions were computed for the two patient groups separately, including item difficulty or discrimination, respectively, as dependent variables. One behavioural (RT in healthy controls) and five psycholinguistic variables (word length, frequency, age of acquisition, semantic diversity, familiarity) were included as independent variables. The two regressions with item difficulty as dependent variable were significant (SD: *F*(6,56) = 13.24, *p* < .001; PSA: *F*(6,56) = 12.52, *p* < .001) and accounted for half of the variance (adjusted *R*^2^ values of .54 and .53 for the SD and PSA, respectively). Inspection of the standardized regression coefficients revealed two significant variables for each analysis. Frequency was a significant variable in both patient regressions (*β*_*SD*_ = −.41, *p* = .003; *β*_*PSA*_ = −.34, *p* = .018), while familiarity was also significant for the SD group (*β*_*SD*_ = −.38, *p* < .001) and word length for the PSA patients (*β*_*PSA*_ = .30, *p* = .009). The absolute standardized regression coefficients are visualized in Fig. 3. To test whether these variables contribute differentially across patient groups, a follow-up regression was computed by adding group as an independent variable and all Group x Item property interaction terms. The only significant interaction effect was Group x Familiarity (*t* = −2.68, *p* = .008), indicating that item familiarity plays a significantly larger role in item difficulty for SD patients compared to PSA patients.

The two regression analyses with item discrimination as the dependent variable explained a negligible portion of variance (adjusted *R*^2^ values of .13 and .05 for the SD and PSA, respectively), and the results of these analyses are thus not reported further in this paper.

## 4 Discussion

We investigated the existence and nature of a systematic gradient of item naming difficulty in semantic dementia and post-stroke aphasia, two neurological conditions with anomia as a central symptom. By employing Item Response Theory, we (a) established that an item naming difficulty gradient exists, which (b) partly differs between patient groups, and is (c) partially related to a limited number of psycholinguistic properties – frequency and familiarity for SD, frequency and word length for PSA.

### 4.1 A systematic gradient of item naming difficulty exists but differs across aetiology

Confirming clinical intuition, our results provide compelling evidence for the presence of a systematic gradient of item naming difficulty in both patient groups. The existence of a gradient means that the probability of successfully naming an item is not only dependent on overall anomia severity but also on the specific item to be named. If we imagine two patients with severe anomia who both name three out of ten pictures correctly, it is probable that they will get the same items correct or incorrect, respectively. The difficulty gradient was, however, not exactly the same between the two patient groups studied here. Approximately half of the items were significantly harder or easier to name for an SD patient *vs* a PSA patient with the same underlying naming ability.

### 4.2 A limited number of psycholinguistic item properties influences item difficulty

Having established that an item naming difficulty gradient exists but partly differs across patient groups, we then explored how strongly the gradient was related to an a priori selection of psycholinguistic variables, and if these relationships varied across aetiologies, as would be expected based on previous research. The regression analyses were significant for SD and PSA and explained over 50% of the variance. Frequency was the variable that related to an item's difficulty in both patient groups, suggesting that words encountered more often are less susceptible to language impairments in general. In PSA patients, word length was the only other significant variable. These findings are largely in line with Fergadiotis et al. (2015), where word length, frequency plus additionally age of acquisition explained 62% of the variance of item naming difficulty in a PSA sample. The relative importance of word length for item difficulty in PSA patients in particular can be explained by (i) commonly occurring phonological impairment in most PSA patients (Halai et al., 2017) which is sensitive to word length (Crisp & Lambon Ralph, 2006; Nickels & Howard, 2004); and (ii) the co-occurring motor speech impairments in many PSA patients with anterior damage (Ziegler et al., 2022).

In SD patients the only additional contributor to item difficulty – over and above frequency – was familiarity. This result is in line with previous research which has found that SD patients' production in naming and connected speech, as well as comprehension are strongly related to a concept's familiarity (Bird et al., 2000; Lambon Ralph et al., 1998; Rogers et al., 2015). There may be at least two underpinning sources of this familiarity effect in SD. Premorbid conceptual representations are stronger when the concept is reinforced more often during learning and thus are more robust (but not immune) to the effects of ATL-centred atrophy (as observed in formal computational models of semantic memory and its decline in semantic dementia (Rogers et al., 2004)). Secondly, even during decline, the patients' ongoing experience may drive partial reinforcement, which will be greatest for the most commonly occurring, familiar concepts. Accordingly, familiarity effects can become augmented during decline (Welbourne et al., 2011).

As for the approximately 50% of the variance in item difficulty that remain unexplained, several factors might be of relevance. First, successful picture naming relies on a multitude of elements, ranging from visually processing the stimulus, to activating the appropriate semantic content and accessing/selecting the correct word form, through to accurate articulation. The psycholinguistic properties included in our analyses only cover a limited number of these required elements, so others (for instance properties of the picture, influencing visual processing) are not considered. Furthermore, impairments in other non-language cognitive functions (such as attention or executive function) or other patient-related factors (such as age and education) might affect item-level performance, and thus influence the estimated item parameters. Finally, all of these elements, both language and non-language, would be subject to individual differences and thus add noise to the data.

Finally, we note that, unlike item *difficulty*, there was no relationship between the item *discrimination* parameter and psycholinguistic properties for either patient group. One possible explanation for this result is that almost all 64 items have high (>1) discrimination values by IRT standards (Baker, 2001) and, in turn, there is little variation amongst these high values and thus no relationship with psycholinguistic properties. These high discrimination values could be related to the distinct clinical signs associated with variability in the latent variable (anomia severity) in contrast to other applications of IRT such as educational research where items are required to discriminate between more subtle differences in the latent variable (Klinkenberg et al., 2011).

### 4.3 Implications, limitations and future directions

Our findings have important implications relating to test interpretation, construction and beyond. First, the existence of a difficulty gradient offers an exciting opportunity to construct potentially more precise, tailored and efficient assessments. For instance, the gradients could be used for adaptive testing procedures whereby probe items of a certain difficulty level are chosen and the next item would depend on the success of naming the previous item. A similar approach could also be used in therapy settings, where the gradient would be helpful for choosing which pool of items would ideally be worked on next and thus constitute the “zone of proximal re-development” (Conroy et al., 2012; Vygotsky, 1978).

Second, the finding of partly different difficulty gradients depending on aetiology generates both challenges and opportunities. Due to the differences between groups, it might be questionable as to whether it is sensible and justified to use the same assessment for different aetiology groups. At least when it comes to interpretation, the same total score might, at worst, not signify a comparable level of anomia severity (or naming ability) in one group *vs* another. Also, different gradients (i.e., a different item order) would have to be used for adaptive test construction. On the other hand, these group differences could potentially also be used to selectively construct tests that may help with differential diagnosis.

Third, knowledge about the factors that contribute most to an item's difficulty could be useful for estimating the difficulty of items that were not part of the studied item pool. This might be useful for the creation of parallel test versions or for an entirely new approach to tests that are constructed to systematically sample the variables in question.

The current investigation was limited to one naming test and two samples of patients. While the sample sizes are large for studies with such patient cohorts, they are on the smaller size for studies employing an IRT approach. As a consequence, our IRT results have higher margins of error compared to other IRT studies, e.g., Pedraza et al. (2011). Also, the PSA sample size was not large enough to explore potential differences across subgroups (e.g., comparing fluent and non-fluent patients).

Future studies should investigate to what extent the current findings hold in other (ideally larger) samples, with a different selection of items, a broader coverage of (psycholinguistic) item properties, and in languages other than English. In doing so it will be worth ensuring a broad coverage of anomia severity in the sample, as was true in the current study for both PSA and SD patients.

## Funding

This research was supported by a grant from the Independent Research Fund Denmark (grant number DFF-1024-00139B) to Randi Starrfelt, an MRC Career Development Award to ADH (MR/V031481/1), and an MRC Programme grant (MR/R023883/1) as well as an intramural award (MC_UU_00005/18) to MALR.

## Open access

For the purpose of open access, the UKRI-funded authors have applied a Creative Commons Attribution (CC BY) licence to any Author Accepted Manuscript version arising from this submission.

## CRediT authorship contribution statement

**Erling Nørkær:** Writing – original draft, Visualization, Methodology, Formal analysis, Conceptualization. **Ajay D. Halai:** Writing – review & editing, Methodology, Investigation, Conceptualization. **Anna Woollams:** Writing – review & editing, Investigation. **Matthew A. Lambon Ralph:** Writing – original draft, Supervision, Project administration, Methodology, Conceptualization. **Rahel Schumacher:** Writing – original draft, Supervision, Project administration, Methodology, Conceptualization.

## Declaration of competing interest

The authors declare no conflicts of interest concerning the research, authorship, or publication of this study.
