# Supplementary material for: Establishing and evaluating the gradient of item naming difficulty in post-stroke aphasia and semantic dementia
Source: Cortex. 2024 Oct;179:103–11. doi: 10.1016/j.cortex.2024.07.007 (PMC11413477; doi:10.1016/j.cortex.2024.07.007)
Supplement: Multimedia component 1 [file mmc1.docx]

**Establishing and evaluating the gradient of item naming difficulty
in post-stroke aphasia and semantic dementia**

Erling Nørkær^1^, Ajay D. Halai^2^, Anna Woollams^3^, Matthew A. Lambon Ralph^2†*^, Rahel Schumacher^2,4†*^

# SUPPLEMENTARY MATERIAL

Table S1 contains all the individual item parameters as well as indicators of how well each item fits the Final Model (item RMSEA and associated *p*-values). Additionally, the table contains information about which items contain DIF (based on the analyses of the differences in item parameters in the Anchored Model), as well as a measure of the effect size and direction of the DIF (Expected Score Standardized Difference (ESSD)). The ESSD is equivalent to a Cohen’s *d* measure of the difference in expected test values between the two patient groups. In general, items fit adequately to the Final Model.

**Table S1. Item parameters for the SD and PSA IRT model.**

| Item | SD IRT Final Model | | | |  | PSA IRT Final Model | | | |  | DIF analyses | |
| --- | --- | --- | --- | --- | --- | --- | --- | --- | --- | --- | --- | --- |
|  | Difficulty parameter | Discrimination parameter | Item fit – RMSEA | Item fit – *p* value |  | Difficulty parameter | Discrimination parameter | Item fit – RMSEA | Item fit – *p* value |  | DIF? | ESSD |
| helicopter | -0.44 | 1.49 | 0.00 | 0.55 |  | 0.95 | 1.68 | 0.11 | 0.07 |  | DIF | -0.46 |
| mouse | -0.15 | 1.41 | 0.00 | 0.68 |  | -0.15 | 1.41 | 0.10 | 0.18 |  | No DIF | 0.05 |
| toaster | 0.70 | 1.47 | 0.00 | 0.83 |  | 0.70 | 1.47 | 0.00 | 0.52 |  | No DIF | 0.09 |
| strawberry | 0.50 | 1.25 | 0.03 | 0.35 |  | 1.52 | 1.78 | 0.07 | 0.23 |  | DIF | -0.49 |
| suitcase | 1.22 | 1.10 | 0.00 | 0.46 |  | 1.22 | 1.10 | 0.10 | 0.07 |  | No DIF | -0.38 |
| cat | -1.64 | 1.12 | 0.05 | 0.11 |  | -1.64 | 1.12 | 0.26 | 0.01 |  | No DIF | -0.05 |
| bicycle | -2.07 | 1.46 | 0.02 | 0.41 |  | -0.88 | 0.72 | 0.06 | 0.25 |  | DIF | -0.38 |
| apple | -0.70 | 0.74 | 0.00 | 0.66 |  | -0.70 | 0.74 | 0.08 | 0.15 |  | No DIF | -0.09 |
| rabbit | 0.08 | 1.29 | 0.06 | 0.12 |  | 0.08 | 1.29 | 0.14 | 0.01 |  | No DIF | -0.34 |
| sledge | 2.24 | 1.56 | 0.04 | 0.29 |  | 1.63 | 0.93 | 0.05 | 0.29 |  | DIF | 0.31 |
| dustbin | -0.71 | 1.37 | 0.04 | 0.18 |  | 0.28 | 0.95 | 0.10 | 0.09 |  | DIF | -0.35 |
| frog | 0.72 | 1.82 | 0.00 | 0.57 |  | 0.33 | 1.08 | 0.10 | 0.09 |  | DIF | 0.06 |
| tomato | 1.80 | 1.23 | 0.01 | 0.43 |  | 1.80 | 1.23 | 0.07 | 0.19 |  | No DIF | -0.18 |
| lorry | -1.08 | 1.33 | 0.04 | 0.26 |  | 1.20 | 1.60 | 0.11 | 0.07 |  | DIF | -0.76 |
| cow | -0.71 | 1.24 | 0.00 | 0.87 |  | -0.71 | 1.24 | 0.02 | 0.36 |  | No DIF | 0.06 |
| watering can | 1.53 | 1.63 | 0.04 | 0.26 |  | 1.53 | 1.63 | 0.14 | 0.02 |  | No DIF | -0.25 |
| pineapple | 1.65 | 1.90 | 0.00 | 0.61 |  | 1.65 | 1.90 | 0.00 | 0.72 |  | No DIF | 0.00 |
| bus | -2.26 | 1.30 | 0.00 | 0.44 |  | 0.13 | 1.23 | 0.05 | 0.30 |  | DIF | -0.58 |
| stool | 1.13 | 1.48 | 0.05 | 0.18 |  | 1.13 | 1.48 | 0.09 | 0.14 |  | No DIF | 0.06 |
| dog | -3.45 | 1.02 | 0.05 | 0.19 |  | -1.10 | 1.18 | 0.07 | 0.25 |  | DIF | -0.46 |
| cherry | 0.95 | 1.06 | 0.00 | 0.68 |  | 0.95 | 1.06 | 0.05 | 0.32 |  | No DIF | -0.29 |
| basket | 1.69 | 1.31 | 0.00 | 0.67 |  | 1.69 | 1.31 | 0.10 | 0.10 |  | DIF | 0.55 |
| train | -1.85 | 1.00 | 0.03 | 0.30 |  | -0.39 | 1.18 | 0.00 | 0.47 |  | DIF | -0.31 |
| squirrel | 1.26 | 1.21 | 0.06 | 0.10 |  | 1.26 | 1.21 | 0.04 | 0.35 |  | No DIF | -0.18 |
| pear | 0.41 | 1.39 | 0.00 | 0.82 |  | 0.41 | 1.39 | 0.00 | 0.79 |  | No DIF | 0.02 |
| horse | -2.00 | 1.56 | 0.00 | 0.44 |  | -0.28 | 1.00 | 0.19 | 0.00 |  | DIF | -0.44 |
| motorbike | -0.84 | 1.21 | 0.00 | 0.66 |  | 1.09 | 1.19 | 0.02 | 0.41 |  | DIF | -0.69 |
| banana | -0.67 | 1.00 | 0.08 | 0.01 |  | 0.40 | 1.03 | 0.10 | 0.10 |  | DIF | -0.34 |
| barrel | 1.91 | 1.58 | 0.00 | 0.67 |  | 1.05 | 0.93 | 0.04 | 0.34 |  | DIF | 0.37 |
| plane | -1.95 | 0.92 | 0.00 | 0.91 |  | 0.46 | 1.79 | 0.16 | 0.04 |  | DIF | -0.54 |
| orange | 1.27 | 0.78 | 0.02 | 0.38 |  | 1.27 | 0.78 | 0.10 | 0.05 |  | No DIF | -0.17 |
| piano | -0.09 | 1.50 | 0.03 | 0.35 |  | -0.09 | 1.50 | 0.10 | 0.11 |  | No DIF | -0.33 |
| tortoise | 1.82 | 2.84 | 0.00 | 0.46 |  | 0.78 | 1.17 | 0.00 | 0.87 |  | DIF | 0.42 |
| pliers | 1.74 | 1.51 | 0.00 | 0.47 |  | 1.74 | 1.51 | 0.08 | 0.19 |  | No DIF | 0.21 |
| key | -1.25 | 1.21 | 0.03 | 0.31 |  | -1.25 | 1.21 | 0.10 | 0.17 |  | No DIF | 0.05 |
| penguin | 1.74 | 1.65 | 0.00 | 0.70 |  | 1.01 | 1.19 | 0.06 | 0.24 |  | DIF | 0.29 |
| axe | 0.71 | 1.12 | 0.00 | 0.81 |  | -0.11 | 0.95 | 0.05 | 0.29 |  | No DIF | 0.28 |
| monkey | 0.67 | 1.35 | 0.00 | 0.76 |  | 0.67 | 1.35 | 0.07 | 0.21 |  | No DIF | 0.06 |
| toothbrush | -0.01 | 1.40 | 0.00 | 0.59 |  | 1.14 | 1.66 | 0.06 | 0.25 |  | DIF | -0.45 |
| eagle | 2.66 | 1.23 | 0.00 | 0.43 |  | 0.94 | 0.77 | 0.04 | 0.33 |  | DIF | 0.89 |
| saw | -0.29 | 1.60 | 0.07 | 0.05 |  | -0.29 | 1.60 | 0.08 | 0.22 |  | No DIF | 0.00 |
| rhino | 1.86 | 1.92 | 0.04 | 0.26 |  | 1.86 | 1.92 | 0.22 | 0.00 |  | No DIF | 0.35 |
| plug | 1.01 | 1.36 | 0.04 | 0.24 |  | 1.01 | 1.36 | 0.13 | 0.03 |  | No DIF | -0.14 |
| chicken | -0.68 | 1.46 | 0.00 | 0.63 |  | 0.57 | 1.02 | 0.05 | 0.30 |  | DIF | -0.45 |
| spanner | 1.24 | 1.39 | 0.00 | 0.99 |  | 1.24 | 1.39 | 0.06 | 0.26 |  | No DIF | -0.03 |
| kangaroo | 1.69 | 1.66 | 0.00 | 0.63 |  | 1.69 | 1.66 | 0.00 | 0.56 |  | No DIF | 0.15 |
| glass | -0.91 | 1.59 | 0.03 | 0.29 |  | 0.77 | 1.46 | 0.06 | 0.26 |  | DIF | -0.52 |
| duck | 0.16 | 1.13 | 0.00 | 0.85 |  | 0.16 | 1.13 | 0.19 | 0.00 |  | No DIF | -0.10 |
| scissors | -0.41 | 1.88 | 0.03 | 0.33 |  | -0.41 | 1.88 | N/A | N/A |  | No DIF | 0.00 |
| camel | 1.26 | 1.61 | 0.00 | 0.96 |  | 1.26 | 1.61 | 0.30 | 0.00 |  | No DIF | 0.56 |
| envelope | 0.07 | 1.09 | 0.00 | 0.80 |  | 1.13 | 1.49 | 0.02 | 0.41 |  | DIF | -0.41 |
| owl | 0.50 | 1.48 | 0.06 | 0.10 |  | 0.50 | 1.48 | 0.18 | 0.01 |  | No DIF | 0.21 |
| paintbrush | 1.85 | 1.39 | 0.00 | 0.46 |  | 1.85 | 1.39 | 0.11 | 0.09 |  | No DIF | -0.66 |
| tiger | 0.67 | 1.37 | 0.06 | 0.14 |  | 0.67 | 1.37 | 0.00 | 0.57 |  | No DIF | -0.13 |
| comb | 0.39 | 1.35 | 0.07 | 0.06 |  | -0.57 | 1.26 | 0.12 | 0.13 |  | DIF | 0.30 |
| swan | 0.62 | 1.67 | 0.00 | 0.92 |  | 0.62 | 1.67 | 0.15 | 0.04 |  | No DIF | 0.00 |
| screwdriver | 1.09 | 1.15 | 0.01 | 0.42 |  | 1.09 | 1.15 | 0.00 | 0.42 |  | No DIF | -0.51 |
| elephant | -0.03 | 1.39 | 0.00 | 0.85 |  | -0.03 | 1.39 | 0.00 | 0.63 |  | No DIF | -0.24 |
| candle | 0.34 | 1.94 | 0.00 | 0.47 |  | 0.34 | 1.94 | 0.00 | 0.47 |  | No DIF | 0.00 |
| ostrich | 2.63 | 1.94 | 0.05 | 0.22 |  | 1.25 | 1.38 | 0.00 | 0.45 |  | DIF | 0.76 |
| alligator | 1.18 | 1.55 | 0.00 | 0.73 |  | 1.18 | 1.55 | 0.00 | 0.91 |  | No DIF | -0.01 |
| brush | -1.45 | 1.74 | 0.00 | 0.85 |  | 0.46 | 1.19 | 0.08 | 0.19 |  | DIF | -0.55 |
| peacock | 2.64 | 1.70 | 0.00 | 0.82 |  | 1.30 | 1.00 | 0.02 | 0.42 |  | DIF | 0.73 |
| hammer | -0.27 | 1.33 | 0.06 | 0.10 |  | -0.27 | 1.33 | 0.11 | 0.14 |  | No DIF | -0.12 |
| Note: The items *pineapple*, s*aw*, s*cissors*, s*wan* and *candle* were used as anchor items in the IRT model.  Item fit RMSEA: Root Mean Square Error of Approximation. Item fit *p* values are associated with χ^2^ tests testing for significant lack of fit.  DIF: Differential Item Functioning.  ESSD: Expected Score Standardized Difference (equivalent to Cohen’s *d* for the difference in expected test scores between patient groups). Negative values indicate that an item is systematically easier for PSA patients than for SD patients. | | | | | | | | | | | | |
